# SecA is required for membrane targeting of the cell division protein DivIVA *in vivo*

**DOI:** 10.3389/fmicb.2014.00058

**Published:** 2014-02-14

**Authors:** Sven Halbedel, Maki Kawai, Reinhard Breitling, Leendert W. Hamoen

**Affiliations:** ^1^Centre for Bacterial Cell Biology, Institute for Cell and Molecular Biosciences, Newcastle UniversityNewcastle upon Tyne, UK; ^2^FG11 Division of Enteropathogenic bacteria and Legionella, Robert Koch InstituteWernigerode, Germany; ^3^Institut für Molekularbiologie, Friedrich-Schiller-UniversitätJena, Germany; ^4^Bacterial Cell Biology, Swammerdam Institute for Life Sciences, University of AmsterdamAmsterdam, Netherlands

**Keywords:** SecA ATPase, DivIVA, cell division, membrane binding, protein localization

## Abstract

The conserved protein DivIVA is involved in different morphogenetic processes in Gram-positive bacteria. In* Bacillus subtilis*, the protein localizes to the cell division site and cell poles, and functions as a scaffold for proteins that regulate division site selection, and for proteins that are required for sporulation. To identify other proteins that bind to DivIVA, we performed an *in vivo* cross-linking experiment. A possible candidate that emerged was the secretion motor ATPase SecA. SecA mutants have been described that inhibit sporulation, and since DivIVA is necessary for sporulation, we examined the localization of DivIVA in these mutants. Surprisingly, DivIVA was delocalized, suggesting that SecA is required for DivIVA targeting. To further corroborate this, we performed SecA depletion and inhibition experiments, which provided further indications that DivIVA localization depends on SecA. Cell fractionation experiments showed that SecA is important for binding of DivIVA to the cell membrane. This was unexpected since DivIVA does not contain a signal sequence, and is able to bind to artificial lipid membranes *in vitro* without support of other proteins. SecA is required for protein secretion and membrane insertion, and therefore its role in DivIVA localization is likely indirect. Possible alternative roles of SecA in DivIVA folding and/or targeting are discussed.

## INTRODUCTION

DivIVA is a conserved protein that functions in processes related to morphogenesis and development in Gram-positive bacteria. In all organisms examined so far, it localizes to the sites of cell division and cell poles (Edwards et al., 2000; Flärdh, 2003; Ramos et al., 2003; Pinho and Errington, 2004; Nguyen et al., 2007; Lenarcic et al., 2009; Ramamurthi and Losick, 2009; Beilharz et al., 2012; Halbedel et al., 2012). In *Bacillus subtilis,* DivIVA is required for the correct placement of the division septum at midcell. In this division site selection process, DivIVA recruits the FtsZ-ring inhibitors MinCD to the cell poles, thereby preventing division to take place near the poles (Edwards and Errington, 1997; Marston et al., 1998). This involves the transmembrane protein MinJ which bridges DivIVA and MinD (Bramkamp et al., 2008; Patrick and Kearns, 2008). In *divIVA* deletion mutants of *B. subtilis*, MinJ is delocalized and so are the MinCD division inhibiting proteins, resulting in a decreased division frequency and minicell formation (Marston et al., 1998; Bramkamp et al., 2008; Gregory et al., 2008; Patrick and Kearns, 2008). Additionally, DivIVA is important for spore formation and genetic competence. During sporulation, it attracts the RacA protein to the most distal sites of the prespore compartment. RacA binds to the origin of the chromosome and helps to transfer the chromosome into the prespore (Ben-Yehuda et al., 2003; Wu and Errington, 2003). In competent *B. subtilis* cells,**DivIVA dependent sequestration of the Maf and ComN proteins is critical for cell division blockage and DNA uptake efficiency, respectively (Briley et al., 2011; dos Santos et al., 2012).

The function of DivIVA in the Gram-positive kingdom is diverse. For example, in *Listeria* *monocytogenes*, a close relative of *B. subtilis*, the protein appears not to be involved in the localization and function of MinCD, but is required for the secretion of virulence-related autolysins (Halbedel et al., 2012). In actinomycetes, such as *Streptomyces, Corynebacterium*, and *Mycobacterium*, DivIVA functions as a scaffold for polar cell wall biosynthetic proteins and intermediate filaments (Flärdh, 2003; Kang et al., 2008; Letek et al., 2008; Xu et al., 2008; Fuchino et al., 2013), and is required for chromosome segregation by polar attachment of origin regions via the ParA and ParB proteins (Donovan et al., 2012; Ginda et al., 2013). Even in cocci, such as *Streptococcus pneumoniae* and *Enterococcus faecalis*, which neither grow filamentously nor sporulate, DivIVA has an important role, and is required for septation and daughter cell separation (Fadda et al., 2007; Rigden et al., 2008).

DivIVA proteins are generally composed of a highly conserved N-terminal lipid binding domain followed by a less conserved C-terminal domain of varying length (Lenarcic et al., 2009). Both domains contain extensive α-helical coiled–coil motifs that are responsible for dimerisation and tetramerisation (Rigden et al., 2008; Oliva et al., 2010; van Baarle et al., 2013). The N-terminus forms a parallel coiled–coil complex, which is capped with two intertwined loops exposing conserved hydrophobic (F17) and positively charged amino acids (K15/R18). Membrane binding of this structure is achieved by inserting the hydrophobic side chains of two phenylalanines into the phospholipid bilayer, and is stabilized by auxiliary electrostatic interactions (Oliva et al., 2010). DivIVA dimers form tetramers through formation of a four helix bundle that involves the conserved ends of the C-terminal domains (Oliva et al., 2010; van Baarle et al., 2013), and these tetramers can form larger oligomeric structures (Muchová et al., 2002; Stahlberg et al., 2004; Oliva et al., 2010). It has been shown that DivIVA binds to cell division sites and to cell poles because the cell membrane is strongly concave at these areas (Lenarcic et al., 2009; Ramamurthi and Losick, 2009). The underlying mechanism by which DivIVA accumulates at negatively curved (concave) membrane areas is unknown, but Monte Carlo simulations have shown that protein complexes, which mutually interact and weakly bind to membranes, tend to cluster at negatively curved membrane regions. This phenomenon has been described as “molecular bridging” (Lenarcic et al., 2009).

To examine whether other proteins might be involved in the targeting of DivIVA, we performed an *in vivo* cross-linking and pull-down experiment. This approach yielded an unexpected candidate; the secretion ATPase SecA. However, DivIVA does not contain a signal sequence. It seems therefore unlikely that SecA would be involved in the activity of DivIVA. However, there are several papers describing *secA* mutants in *B. subtilis* that inhibited sporulation. Since DivIVA is essential for sporulation we were curious whether something might be happening with DivIVA in these mutants. Interestingly, the localization of DivIVA was strongly disturbed in these *secA* mutants, and it appears that SecA is required for membrane targeting of DivIVA *in vivo*.

## MATERIALS AND METHODS

### BACTERIAL STRAINS AND GROWTH CONDITIONS

The *B. subtilis* strains used in this study are listed in Table 1. Routinely, cells of *B. subtilis* were cultivated either in Luria Bertani (LB) broth or Spizizen minimal medium (SMM) (Anagnostopoulos and Spizizen, 1961) supplemented with 0.5% glucose, 20 μg/ml L-tryptophan, 6 mM MgSO_4_, 0.02% casein hydrolysate and 0.0011% ammonium iron(III) citrate. If necessary, antibiotics and additional supplements were added at the following concentrations: erythromycin (1 μg/ml), chloramphenicol (5 μg/ml), spectinomycin (50 μg/ml), tetracycline (10 μg/ml), IPTG (1 mM) or xylose (0.5–2.0%). For all cloning procedures *Escherichia coli* DH5α was used as the standard plasmid host, whereas *E. coli* BL21(DE3) was used for protein overexpressions (Sambrook et al., 1989). Plasmids that had to be inserted into the *B. subtilis* chromosome by single crossover were passaged through the *recA*^+^ *E. coli* strain MC1061 (Casadaban and Cohen, 1980) prior to their transformation into different *B. subtilis* recipients.

**Table 1:** *B. subtilis* strains and plasmids used in this work.

Strain/plasmid	Relevant characteristics	Source*/reference
*Bacillus subtilis* strains
168	*trpC2*	lab collection
AZ1	*trpC2 secAT128A*	Nakane et al. (1995)
I*secA*	*trpC2 secA′ erm Pspac-secA*	Jongbloed et al. (2002)
NIG1121	*metB101 hisH101*	Sadaie and Kada (1985)
NIG1152	*metB101 hisH101 secA341*	Sadaie and Kada (1985)
1803	*trpC2* Ω*divIVA::(PdivIVA-divIVA-gfp cat)*	Thomaides et al. (2001)
3308	*trpC2* Ω*divIVA::*(*PdivIVA-divIVA-gfp-his6 erm*)	pQEDG1 → 168
4041	*trpC2* Δ*divIVA::tet*	Oliva et al. (2010)
4066	*trpC2* Ω*amyE::(Pxyl-divIVA-gfp spc)*	pDG9→168
4067	*trpC2* Δ*divIVA::tet*Ω*amyE::(Pxyl-divIVA-gfp spc)*	4066→4041
4072	*trpC2* Δ*divIVA::tet*Ω*amyE::(Pxyl-divIVA-gfp spc) secA′ erm Pspac-secA*	I*secA*→4067
4097	*trpC2* Ω*amyE::*(*PdivIVA-gfp-his10 erm*)	pQEG2→168
BSN3	*trpC2* Ω*amyE::(Pxyl-divIVA-gfpA206K spc)*	pSH3→168
BSN47	*trpC2* Ω*amyE::(Pxyl-divIVA spc) secA′ erm Pspac-secA*	pSH19→I*secA*
BSN49	*trpC2* Ω*amyE::(Pxyl-divIVA spc)*	pSH19→168
BSN50	*trpC2* Ω*amyE::(PdivIVA*-*his6-divIVA spc)*	pSH18→168
BSN51	*trpC2* Δ*divIVA::tet *Ω*amyE::(Pxyl-divIVA spc)*	4041→BSN49
BSN52	*trpC2* Δ*divIVA::tet *Ω*amyE::(Pxyl-divIVA spc) secA′ erm Pspac-secA*	4041→BSN47
BSN61	*metB101 hisH101* Ω*amyE::(Pxyl-divIVA-gfpA206K spc)*	BSN3→NIG1121
BSN62	*metB101 hisH101 secA341* Ω*amyE::(Pxyl-divIVA-gfpA206K spc)*	BSN3→NIG1152
BSN66	*metB101 hisH101* Δ*divIVA::tet *Ω*amyE::(Pxyl-divIVA-gfpA206K spc)*	4041→BSN61
BSN67	*metB101 hisH101 secA341* Δ*divIVA::tet *Ω*amyE::(Pxyl-divIVA-gfpA206K spc)*	4041→BSN62
BSN73	*trpC2* Ω*amyE::(PdivIVA*-*gfp-his6 erm)*	pSH34→168
BSN77	*trpC2* Δ*divIVA::tet* Ω*amyE::(Pxyl-divIVA-gfp spc) secY′ cat Pspac-secY*	pDY6→4067
BSN131	*trpC2* Ω*secA::(Pspac*-*secA*^2141-2536^-his12 erm)	pSH68→168
BSN158	*trpC2* *secA′ erm Pspac-secA ΩaprE::(PdivIVA-divIVA-gfp spc)*	pSH84→I*secA*
BSN164	*trpC2 secA′ erm Pspac-secA ΩaprE::(PdivIVA-divIVA-gfp spc) ΩamyE::(PsecA-secA12 cat)*	pSH98→BSN158
BSN226	*trpC2 secAT128A *Ω*amyE::(Pxyl-divIVA-gfp spc)*	pDG9→AZ1
BSN228	*trpC2 secAT128A *Ω*amyE::(Pxyl-divIVA-gfp spc)* Δ*divIVA::tet*	4041→BSN226
**Plasmids**
pAPNC213	*bla aprE5′ spc lacI aprE3^′*	Morimoto et al. (2002)
pDY6	*bla Pspac-secY lacI cat*	Breitling et al. (1994)
pMUTinHis	*bla erm Pspac-his12 lacI*	Ishikawa et al. (2006)
pX	*bla amyE5′* xylR cat amyE3^′	Kim et al. (1996)
pDG7	*bla amyE3′* spc Pxyl-PdivIVA-divIVA-gfp amyE5^′	Lenarcic et al. (2009)
pDG9	*bla amyE3′* spc Pxyl-divIVA-gfp amyE5^′	Oliva et al. (2010)
pDG24	*bla amyE3′* spc Pxyl-PdivIVA-his6-divIVA-gfp amyE5^′	this work
pQEDG1	*bla divIVA-gfp-his6 erm*	Lenarcic et al. (2009)
pQEG2	*bla PdivIVA-gfp-his10 ′*amyE^′ erm	Lenarcic et al. (2009)
pSH3	*bla amyE3′* spc Pxyl-divIVA-gfpA206K amyE5^′	Oliva et al. (2010)
pSH18	*bla amyE3′* spc Pxyl-PdivIVA-his6-divIVA amyE5^′	this work
pSH19	*bla amyE3′* spc Pxyl-divIVA amyE5^′	this work
pSH34	*bla PdivIVA-gfp-his6 ′*amyE^′ erm	this work
pSH68	*bla erm Pspac-secA*^2141-2536^-his12 lacI	this work
pSH83	*bla amyE5′* PsecA-secA cat amyE3^′	this work
pSH84	*bla aprE5′* spc lacI PdivIVA-divIVA-gfp aprE3^′	this work
pSH98	*bla amyE5*′* PsecA-secA12 cat amyE3*′	this work

*The arrow (→) indicates a transformation where the donor and the recipient are given on the left and the right side of the arrow, respectively.

### GENERAL METHODS, DNA MANIPULATION, AND OLIGONUCLEOTIDES

Transformation of *E. coli* and plasmid DNA extraction were performed according to standard protocols (Sambrook et al., 1989). Transformation of *B. subtilis* was done as described previously (Hamoen et al., 2002). Enzymatic DNA manipulations and modifications were carried out according to the information given by the manufacturer’s guidelines. For site directed mutagenesis of plasmid DNA, a modified Quickchange mutagenesis protocol was employed (Zheng et al., 2004). All oligonucleotides used in this study are listed in Table 2.

**Table 2:** Oligonucleotides used in this study.

Name	Sequence (5′→3′)
div25	CCATTAACGCCAAATGATATTCACA
xyl4	CATCCTAGGAATCTCCTTTCTAG
LH93	ACACAATCTAAACTTTCCAAAGATCCC
LH94	TTTGGAAAGTTTAGATTGTGTGGACAG
LH102	TCACCACGGAGGCATGCCATTAACGCCAAATGA
LH103	TGATGGTGATGTCCCATGATGCCACCTCCATTTTTAC
SV23	GAAAAGGAATAACTTGATATCGAATTC
SV24	GATATCAAGTTATTCCTTTTCCTCAAATAC
SV62	CACCATCACCATCACTAACATCACCATTAAGCTTAATTAG
SV63	CTTAATGGTGATGTTAGTGATGGTGATGGTGATGAGATC
SV96	GCTCGAGTTCAGTACGGCCGCAGC
SV97	GCGGTCGACGATGTTCTCCGCCAGCAG
SV116	GACTCTAGACCGTGATGTCCGCGGAAGG
SV117	GACTCTAGAGTAAACTTGCCGGGGCGAAC
SV140	CATGAACTACGCCGGATTGACAATCAG
SV141	CAATCCGGCGTAGTTCATGTCGTTCTG

### CONSTRUCTION OF STRAINS CONTAINING ECTOPICALLY EXPRESSED VARIANTS OF *div*IVA

Plasmid pDG7 (Lenarcic et al., 2009) expresses the *PdivIVA-divIVA-gfp* allele, and was used for the insertion of *divIVA-gfp* into the *amyE* locus of *B. subtilis*. Plasmid pDG9 in turn contains *divIVA-gfp* driven by the xylose-inducible *Pxyl* promoter. For its construction the *PdivIVA *promoter that is still present in plasmid pDG7 was removed by PCR using the oligonucleotides xyl4/div25 (for all primer sequences see Table 2). To introduce the A206K mutation, which prevents green fluorescent protein (GFP) dimerisation (Zacharias et al., 2002), into the *gfp* part of the *divIVA-gfp* fusion encoded by plasmid pDG9, Quickchange mutagenesis was applied to this vector using LH93/LH94 as the mutagenic primers. The resulting plasmid was sequenced and named pSH3.

In order to obtain a plasmid coding for *his6-divIVA* under the control of the *PdivIVA* promoter, the TAA stop codon was introduced immediately after the end of the *divIVA* part of *PdivIVA-his6-divIVA-gfp* present on plasmid pDG24, using Quickchange mutagenesis and the primer pair SV23/SV24. The obtained plasmid was sequenced and named pSH18. Plasmid pDG24 in turn was obtained by PCR via the introduction of six histidine codons at the 5′-end of the *divIVA-gfp* open reading frame of plasmid pDG7 using primers LH102/LH103. Plasmid pSH19, allowing *Pxyl* controlled expression of the *divIVA* gene from the *amyE* locus of *B. subtilis*, was constructed by site directed mutagenesis of plasmid pSH3. The TAA stop codon was inserted in between the *divIVA* and *gfp* part of the *divIVA-gfp* fusion in the same way as described for plasmid pSH18.

Plasmid pQEG2 is a pQE60 derivative expressing *gfp-his10* controlled by the *divIVA* promoter and contains the *erm* erythromycin resistance marker (Lenarcic et al., 2009). Plasmid pSH34 was obtained from plasmid pQEG2 by truncating the Histag of *gfp-his10* by transforming the seventh histidine codon into a TAA stop codon. To this end, pQEG2 was used as the template in a Quickchange polymerase chain reaction using the oligonucleotides SV62 and SV63. Plasmid pQEDG1 is a derivative of pQE60 and contains the *erm* marker and a* PdivIVA-divIVA-gfp-his6* allele (Lenarcic et al., 2009).

Plasmids that were designed to insert into the *amyE* gene, either by Campbell-type integration (pQEG2, pSH34) or double crossover (pDG9, pSH3, pSH18, and pSH19), were transformed to *B. subtilis*, and amylase negative clones were selected based on iodine staining of starch containing agar plates. Plasmid pQEDG1 was inserted into the *divIVA* locus of the wild type strain 168 in a single crossover recombination event.

In order to obtain a *divIVA-gfp* variant constitutively expressed from the *aprE* locus, the 1.4 kb *Xba*I/*Kpn*I fragment of pDG7 containing the *PdivIVA-divIVA-gfp* allele was cloned into pAPNC213 (Morimoto et al., 2002) digested with the same enzymes. The resulting plasmid was named pSH84, and inserted into the *aprE* locus of strain I*secA* by double cross over. The insertional events were confirmed by PCR.

### CONSTRUCTION OF SecA AND SecY DEPLETION STRAINS

For the depletion of SecA we made use of the *Pspac-secA* allele present in strain I*secA *(Jongbloed et al., 2002), and combined this allele with strain 4067. For depletion of SecY, plasmid pDY6 (Breitling et al., 1994), containing the first 808 N-terminal nucleotides of the *secY* structural gene under control of the *Pspac* promoter, and the *lacI* gene, was inserted at the *secY* locus of strain 4067. As the result of this insertion, a C-terminally truncated fragment of *secY* under control of *PsecY* is left over, whereas transcription of the full-length *secY* gene is induced by the addition of IPTG. The correct insertion of plasmid pDY6 was verified by PCR analysis.

### CONSTRUCTION OF A STRAIN EXPRESSING SecA-His

Plasmid pSH68 was constructed to fuse a 3′-end *his12* tag to the chromosomally encoded *secA* gene by Campbell-type integration. For this purpose a DNA fragment encompassing the last 732 nucleotides of the *secA* open reading frame (except the stop codon) was PCR amplified using the oligonucleotides SV97/SV96, with SV96 as the reverse primer introducing a *Xho*I site at the 3′-end. The obtained fragment was digested by *Eco*RI/*Xho*I (thereby making use of the most C-terminal *Eco*RI site that is present inside the *secA* ORF), and ligated to pMUTinHis (Ishikawa et al., 2006) digested with the same enzymes. The wildtype sequence of the insert was confirmed and the plasmid was transformed to *B. subtilis* 168. Clones were selected on agar plates containing erythromycin, and the correct insertion of the plasmid at the *secA* locus was verified by PCR analysis.

### CONSTRUCTION OF A STRAIN CONTAINING AN ECTOPICALLY EXPRESSED *sec*A12 ALLELE

A DNA fragment containing *PsecA-secA* was PCR amplified from chromosomal DNA using the oligonucleotides SV116 and SV117, digested with *Xba*I, and subsequently ligated with the *Xba*I digested plasmid backbone of plasmid pX devoid of the *xylR* containing fragment. The resulting plasmid was sequenced and named pSH83. In order to introduce the *secA12* mutation (S515L) into the *secA* gene of plasmid pSH83, a Quickchange mutagenesis reaction using oligonucleotides SV140 and SV141 was carried out resulting in plasmid pSH98. Plasmids pSH83 and pSH98 were inserted into the *amyE* gene of strain BSN158 to give strains BSN159 and BSN164, respectively.

### PULL-DOWN ANALYSIS AND PROTEIN IDENTIFICATION

Formaldehyde *in vivo* cross-linking of protein complexes and their subsequent purification by affinity chromatography was carried out as described recently (Ishikawa et al., 2006). Pull-down eluates were either directly analyzed by Western blotting or, where necessary, concentrated in a centrifugation step using Microcon YM-100 centrifugal filter units. To reverse the covalent cross-links, the sample aliquots were heated at 95°C for at least 1 h before they were subjected to electrophoresis. For the identification of putative binding partners of DivIVA-GFP-His, pull-down eluates of strain 3308 were separated by SDS-PAGE. Bands of interest were excised from the gel and analyzed by mass spectrometry.

### ISOLATION OF MEMBRANE FRACTIONS AND WESTERN BLOTTING

Fractionation of *B. subtilis* cells was done as described previously (Blencke et al., 2006). Briefly, cells of a 50 ml culture were harvested and disrupted using sonication in 1 ml buffer M (50 mM Na_2_HPO_4_ 50 mM NaH_2_PO_4_). The total lysate was cleared from debris in a tabletop centrifuge run, and subsequently, membranes were collected by ultracentrifugation (250,000 × *g*, 30 min, 4°C). The supernatant was considered as the cytoplasmic fraction. The membrane pellet was washed three times in buffer M and a fourth time in buffer M containing 600 mM NaCl, before it finally was resuspended in 100 μl buffer M, resulting in a 10-fold concentration. SDS-PAGE and Western blotting were carried out using standard protocols and rabbit antisera recognizing DivIVA (Marston et al., 1998), GFP (lab stock), SecA (Meens et al., 1993) or SecY (this work).

### ANTIBODY PRODUCTION

The anti-SecY antiserum was raised in rabbits against the oligopeptide YAKGTGRSPAGGGQS (custom synthesized by NeoSystems, Strasbourg, France) corresponding to amino acids 245–259 of *B. subtilis* SecY (4th cytoplasmic domain).

### MICROSCOPIC TECHNIQUES

For fluorescence microscopy of living cells, a small volume (0.3 μl) from an exponentially growing culture was mounted onto a microscope slide covered with a thin film of 1% agarose (dissolved in water). Membrane staining was performed using nile red. Images were taken with a Zeiss Axiovert 200M microscope coupled to a CoolsnapHQ CCD camera and processed using the Metamorph software package (Universal Imaging). A previously described protocol was used for the visualization of DivIVA localization by immunofluorescence microscopy (Marston et al., 1998).

## RESULTS

### SecA PULL-DOWN WITH DivIVA

**FIGURE 1:**
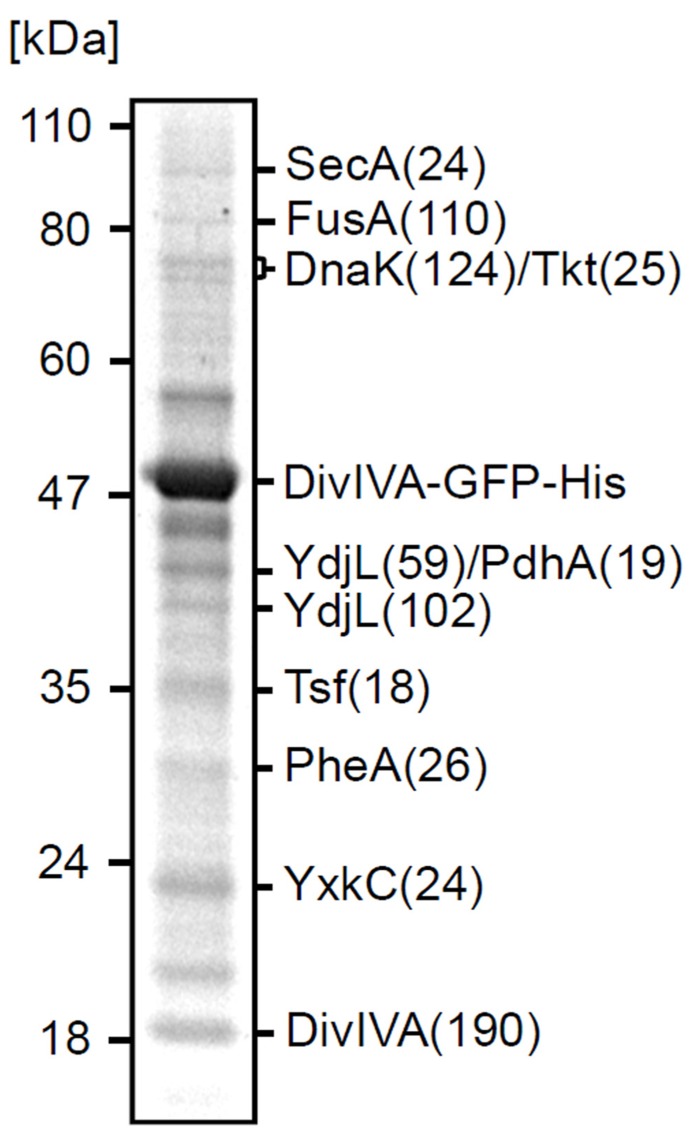
** Cross-linking of SecA and DivIVA**. Separation by SDS-PAGE of the pull-down fraction obtained with DivIVA-GFP-His as bait. The identity of protein bands and the molecular weight marker are indicated. Total score values (Mascot Scores) of the protein identifications are indicated in brackets.

To identify proteins involved in the localization of DivIVA, an *in vivo* cross-linking experiment was performed using a *B. subtilis* strain that expresses a DivIVA-GFP-His-tag fusion (strain 3308). GFP was included to ensure that the His-tagged fusion protein localizes properly. An exponentially growing culture was incubated with 1% formaldehyde for 20 min, followed by the addition of 75 mM glycine to quench the cross-linking reaction. Cells were broken and treated with a Urea/SDS mixture, and the fusion protein was purified on a Ni-NTA affinity column. After reversing the cross-linking by heating, proteins were separated on a SDS-PAA gel (**Figure 1**). Protein bands were identified using mass spectrometry, and corresponded to: elongation factors and a cytosolic chaperone (FusA, Tsf, DnaK), metabolic enzymes (Tkt, PdhA, YdjL, PheA), the unknown protein YxkC, and the secretion ATPase SecA. With the exception of YxkC, the prephenate dehydratase PheA, and SecA, all the other proteins belong to the 100 most abundant cytosolic proteins in exponentially growing *B. subtilis* cells and were therefore considered a non-relevant by-catch (Eymann et al., 2004). We have not found the four known DivIVA binding proteins RacA, MinJ, Maf, and ComN in this experiment. This can be explained by the fact that *racA* and *maf* are only expressed in sporulating or competent cells (Molle et al., 2003; Wu and Errington, 2003; Briley et al., 2011). MinJ contains six multiple transmembrane helices (van Baarle and Bramkamp, 2010) that might complicate its solubilization, and ComN is a small 11.5 kDa protein possibly escaping detection. SecA, however, was a peculiar finding since DivIVA has no signal sequence.

### ABERRANT DivIVA LOCALIZATION IN SPORULATION MUTANT *sec*A12 and *sec*A341

**FIGURE 2:**
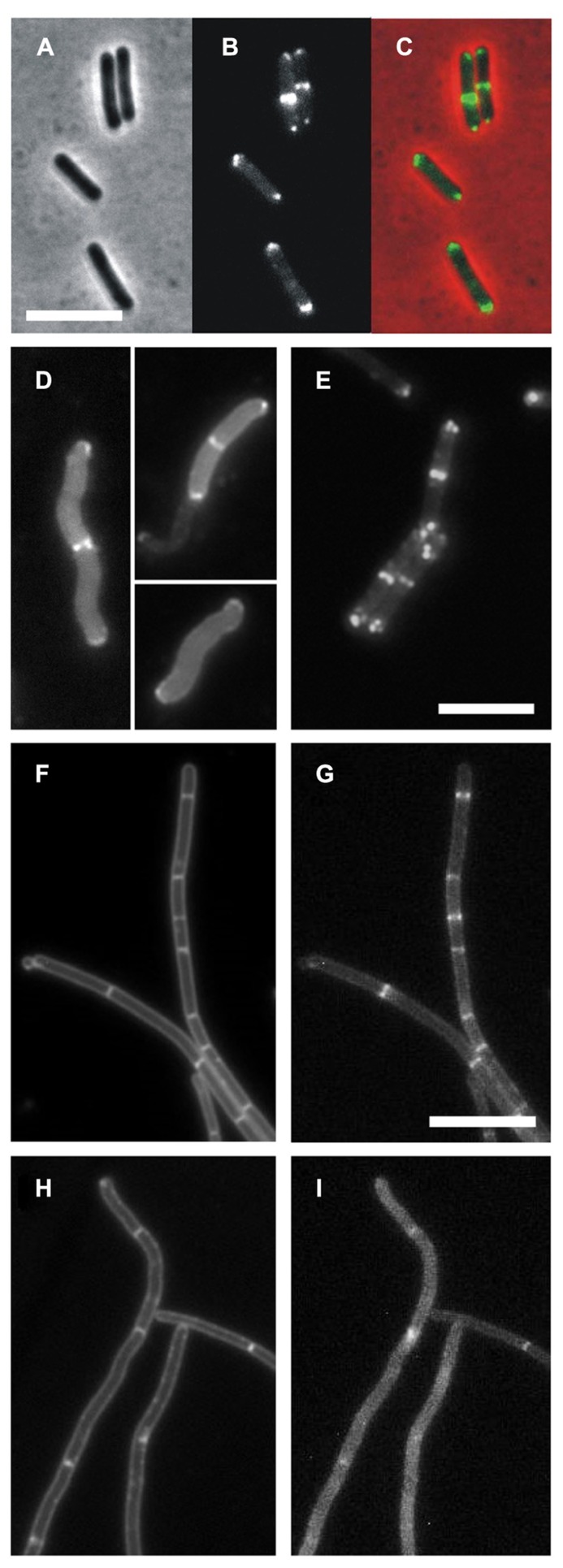
** DivIVA-GFP localization is disturbed in a *secA12* and *secA341* mutant strains. (A–C)** Localization of DivIVA-GFP in wild type *B. subtilis*. Cells of strain 1803 were analyzed by phase contrast **(A)**, and fluorescence microscopy **(B)**. A merged image is shown in **C**. **(D)** Fluorescence micrograph showing the localization of DivIVA-GFP in a strain expressing the SecA12 mutant protein (BSN164, grown without IPTG). **(E)** Same strain with 1 mM IPTG using identical exposure times. **(F–I)** Delocalization of DivIVA-GFP in the *secA341* mutant. An ectopic version of DivIVA-GFP was induced in BSN67 cells containing the temperature sensitive *secA341* mutation and analyzed by fluorescence microscopy during growth at 30°C **(G)**, and at 37°C **(I)**. Nile red stained images are presented in the left panels **(F,H)**. Scale bars are 5 μm for all images. Note that DivIVA-GFP only partially complements the Δ*divIVA* phenotype, explaining filamentation of BSN67 cells.

Asai et al. (1997) described a *secA* mutation (*secA12;* SecA-S515L) that caused an asporogenic phenotype. This sporulation phenotype shows some resemblance to certain previously described *divIVA* mutants (Asai et al., 1997; Thomaides et al., 2001). Since we found SecA in a DivIVA pull-down experiment, we were curious whether a *secA12* mutation might actually affect the localization of DivIVA. The *secA12* mutant is prone to suppressor mutations in *secY* (Kobayashi et al., 2000). To prevent this complication, we constructed a strain containing an IPTG-inducible wild type *secA* gene and a constitutively expressed *secA12* allele at the ectopic *amyE* locus (strain BSN164). In normal cells, DivIVA-GFP localizes at cell division sites and cell poles as shown in **Figures 2A–C** (strain 1803). However, when the *secA12* mutant strain BSN164 was grown without IPTG and only expressed SecA12, the majority of the fluorescent DivIVA-GFP signal remained in the cytoplasm (**Figure 2D**). In the presence of IPTG, BSN164 cells showed normal localization of DivIVA-GFP without any obvious cytoplasmic fluorescence (**Figure 2E**).

More than 25 years ago Sadaie and Kada (1985) isolated the temperature sensitive *secA341* mutant (SecA-P431L). At 37°C this mutant is viable but it shows strongly reduced sporulation efficiencies when compared to growth at the permissive temperature (30°C). To test whether the localization of DivIVA-GFP is disturbed at the non-permissive temperature, *divIVA* was replaced by a xylose-inducible *divIVA-gfp* fusion. The resulting strain (BSN67) was grown at either 30 or 37°C until mid-log growth phase, when DivIVA-GFP expression was induced with 0.5% xylose for 2 h. As shown in **Figures 2H,I**, growth at 37°C resulted in a diffuse GFP signal and irregular fluorescent patches. The diffuse GFP signal was observed in all *secA341* cells grown at 37°C. In contrast, DivIVA-GFP localization in strain BSN67 was normal at the permissive temperature (**Figures 2F,G**), or in wild type cells at 37°C (strain BSN66, data not shown). Importantly, Western blot analysis indicated that the DivIVA-GFP fusion was not affected in the *secA341* mutant at 37°C (data not shown), thus the diffuse fluorescence signal is not a consequence of possible proteolytic degradation at the non-permissive temperature.

### PULL-DOWN CONTROLS

**FIGURE 3:**
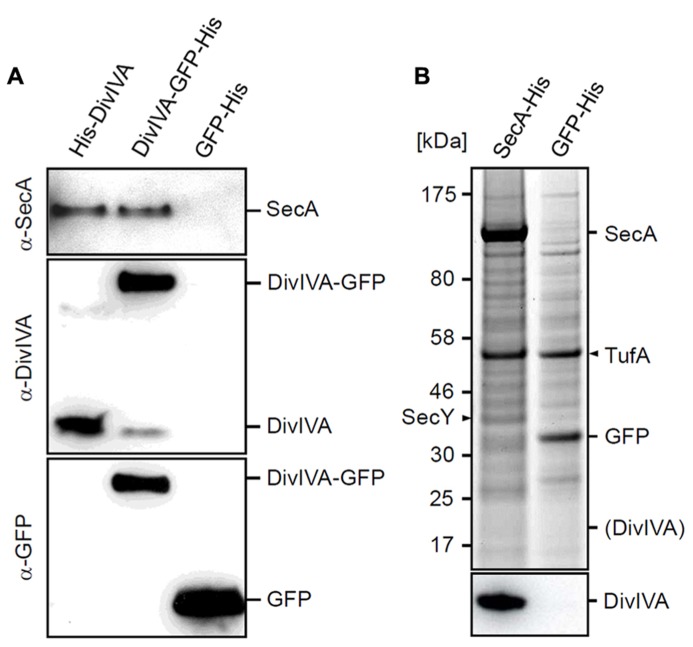
** Control experiments to confirm the cross-linking of SecA with DivIVA. (A)** Pull-down experiment with His-DivIVA, DivIVA-GFP-His and GFP-His as bait proteins (strains BSN50, 3308 and BSN73, respectively). Pull-down fractions were analyzed by Western blotting for the presence of SecA (upper panel). To demonstrate that the bait proteins were pulled down efficiently, fractions were analyzed by Western blotting using DivIVA (middle panel) and GFP antisera (lower panel). **(B)** Pull-down experiment using SecA-His and GFP-His as bait proteins (strains BSN131, and 4097, respectively). Protein fractions were separated by SDS-PAGE (upper panel), and the presence of DivIVA was analyzed by Western blotting (lower panel). SecY, a known interaction partner of SecA, was pulled down with SecA-His. The protein band at around 55 kDa is elongation factor Tu (TufA), which appears to be an unspecific by-catch. The theoretical position of DivIVA on the SDS PAGE gel is indicated in brackets.

The results with the *secA* mutants suggested that the initial pull-down experiment might reveal a biological relevant link between DivIVA and SecA. To corroborate this, a more systematic cross-linking and pull-down analysis was performed using strains that express a His-DivIVA fusion (BSN50), DivIVA-GFP-His fusion (3308), or a GFP-His fusion (BSN73). In this case, the relevant proteins were detected using Western blotting. As shown in **Figure 3A**, SecA was only detected in pull-down eluates with His-DivIVA or DivIVA-GFP-His expressing cells, but not with cell extracts form GFP-His expressing cells. If the interaction between SecA and DivIVA is specific, a reciprocal pull-down experiment, using His-tagged SecA (strain BSN131) as bait, should yield DivIVA. Indeed, as demonstrated in **Figure 3B** a strong DivIVA band was obtained with extracts from cells expressing SecA-His, but not with extracts from cells expressing GFP-His-tag as bait. As fusion of a C-terminal His-tag to the *secA* gene in strain BSN131 did not affect viability, the SecA-His fusion has to be regarded a functional protein. Functionality of SecA-His is further confirmed by the observation that it pulls down its cognate interaction partner SecY (**Figure 3B**).

### SecA IS REQUIRED FOR DivIVA LOCALIZATION

**FIGURE 4:**
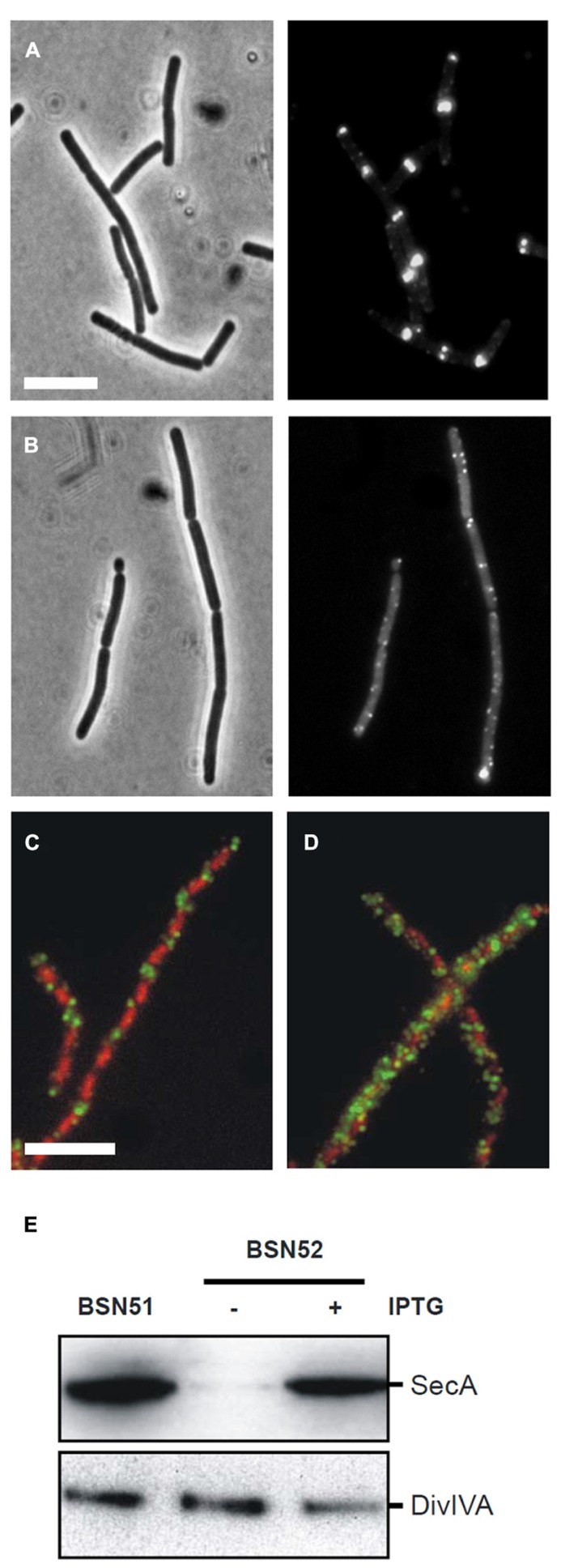
** Effect of SecA depletion on localization of DivIVA-GFP. (A,B)** Localization of DivIVA-GFP in the *secA* depletion strain 4072 grown in the presence of 1 mM IPTG **(A)** or in the absence of IPTG **(B)**. The corresponding phase contrast images are presented in the left panels. **(C,D)** Localization of untagged DivIVA after SecA depletion. Localization of DivIVA in the *secA* depletion strain BSN52 grown with 1 mM IPTG **(C)** or without the inducer **(D)**. DivIVA localization was analyzed by immunofluorescence microscopy using the DivIVA antiserum 2 h after induction with 0.5% xylose. Note that longer induction times would be required for full complementation of the filamentous Δ*divIVA* phenotype. Merged images are composed of red DAPI stained nucleoids and green DivIVA. Depletion of SecA and normal DivIVA expression was confirmed by Western blot analysis **(E)**.

The-pull-down and *secA* mutant experiments suggested that localization of DivIVA requires SecA. To follow delocalization of DivIVA-GFP in time, an IPTG-inducible *Pspac-secA* allele was employed that allows for conditional *secA* expression (Jongbloed et al., 2002). It took about 5 h (~ three generations in SMM medium at 30°C) to notice depletion of SecA (verified by Western blotting), and related growth retardation. However, at this point the localization of DivIVA-GFP looked normal (not shown). The reason for this discrepancy could be the strong self-interaction of DivIVA. DivIVA forms octamers that assemble into large protein clusters (Stahlberg et al., 2004; Oliva et al., 2010), and it is likely that DivIVA-GFP synthesized during the SecA depletion will bind to DivIVA that has been normally deposited when SecA was still present. To circumvent this problem we deleted the wild type copy of *divIVA*, and used a xylose-inducible DivIVA-GFP fusion (strain 4072). When SecA was depleted in this strain, expression of DivIVA-GFP was induced for 2 h with 0.5% xylose. Under these conditions the DivIVA-GFP signal became spotty and diffuse, and no septal or polar localized DivIVA was observed (**Figure 4B**), while it was normal in the presence of IPTG (**Figure 4A**). Western blot analysis confirmed that SecA was indeed depleted, and that there was no degradation of DivIVA-GFP (not shown). To eliminate the possibility that these results were somehow a consequence of the GFP tag, we analyzed the localization of untagged wild type DivIVA using immunofluorescence microscopy. In this experiment a *Pspac-secA* strain was used that contained a xylose-inducible *divIVA* gene (BSN52). Although the resolution of immunofluorescence labeling is poor compared to GFP labeling, in normal cells a regular fluorescence band pattern is observed that is restricted to areas between the nucleoids (**Figure 4C**), as has been shown before (Thomaides et al., 2001). When DivIVA was induced after SecA depletion, a spotty and dispersed fluorescent signal was obtained, clearly indicating that DivIVA was delocalized (**Figure 4D**). Again, as shown in the Western blot of **Figure 4E**, this result was not due to a possible degradation of DivIVA.

### SecA AFFECTS MEMBRANE BINDING OF DivIVA

**FIGURE 5:**
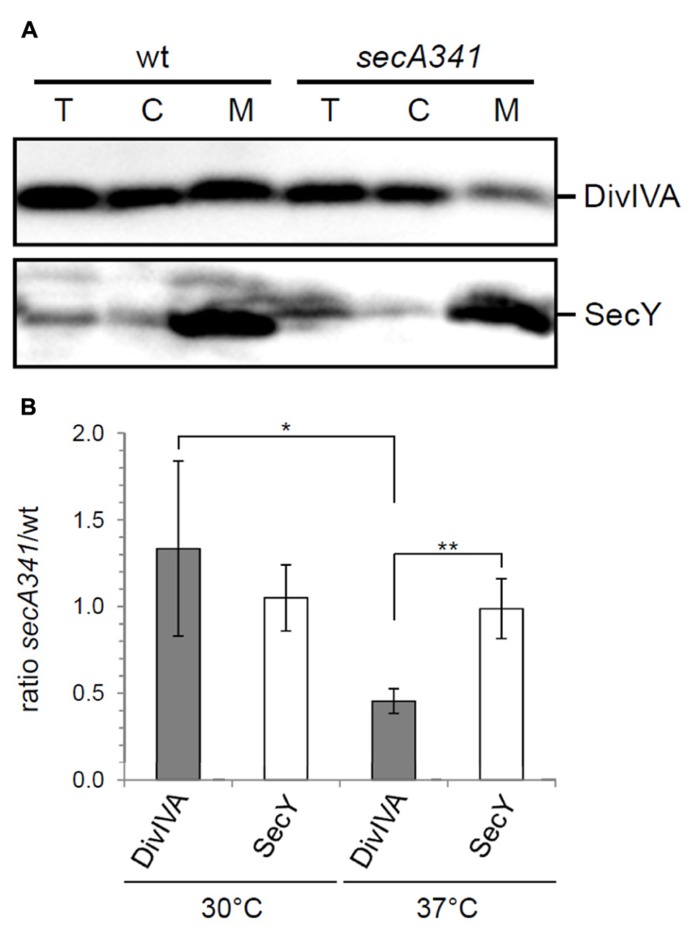
** SecA affects membrane targeting of DivIVA. (A)** Distribution of DivIVA in membrane and cytoplasmic fractions of wild type cells (strain NIG1121), and in cells of the temperature sensitive *secA341* mutant (strain NIG1152) during growth at 37°C. DivIVA concentrations were determined by Western blotting (upper panel), and a parallel Western blot using an antiserum directed against SecY was included as a control (lower panel, T – total extract, C – cytoplasm, M – membrane). Note that membrane samples have been concentrated 10-fold in the course of their isolation. **(B)** Densitometric quantification of Western blot signals from four independent experiments are shown in panel **A**. Values are given as ratios of DivIVA and SecY levels detected in the membrane fraction of *secA341* cells relative to their levels in wild type cells at the indicated temperatures. Asterisks are used to indicate significance levels: *P *< 0.05 (*) or *P *< 0.005 (**).

DivIVA binds to lipid membranes and after cell fractionation a noticable amount of DivIVA ends up in the membrane fraction (Wang et al., 2009). The diffuse fluorescence signals in the *secA* mutants suggest that targeting of DivIVA to the membrane is affected. To test this, wild type strain NIG1121 and *secA341* mutant strain NIG1152 were grown at 30 and 37°C, and the cytosolic and membrane protein fractions were isolated and analyzed by Western blotting. There was no difference in the amount of DivIVA in the membrane fractions of the two different strains when grown at 30°C (data not shown). However, at 37°C the *secA341* cells contained clearly less DivIVA in the membrane fraction compared to wild type cells, even though the total amount of DivIVA was not reduced in both strains (**Figure 5A**, upper panel). Densitometric quantification revealed that the amount of DivIVA present in the membrane fraction of *secA341* cells dropped down to 45 ± 7% of the wild type level during growth at 37°C (**Figure 5B**). This suggests that SecA is required for targeting of DivIVA to the cell membrane. A parallel Western blot with antiserum against SecY was used as a loading control (**Figure 5A**, lower panel). The insertion of SecY into the membrane is not reduced in the *secA341* mutant at 37°C (99 ± 17% of wild type level, **Figure 5B**), which is in agreement with a previous report demonstrating that blockage of protein secretion in the *secA341* mutant requires cultivation at temperatures above 40°C (Leloup et al., 1999).

### LOCALIZATION OF DivIVA IS DISTURBED BY AZIDE

**FIGURE 6:**
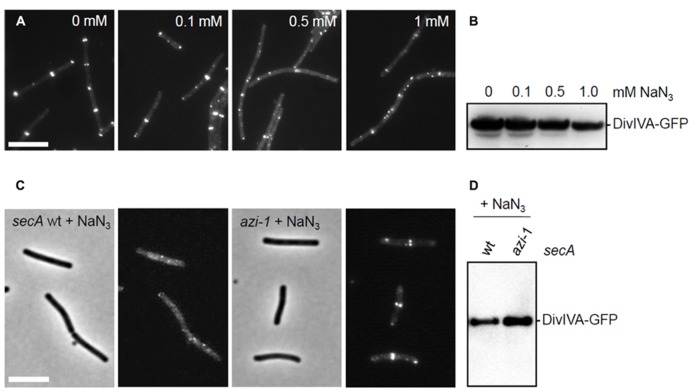
** Effect of azide on the localization of DivIVA-GFP**. **(A)** Fluorescence micrographs showing the localization of DivIVA-GFP in strain 4067 pretreated with increasing amounts of sodium azide. **(B,D)** Western blot analyses to assess the stability of DivIVA-GFP during the sodium azide treatments. **(C)** DivIVA-GFP localization is not disturbed by 0.5 mM sodium azide in cells expressing an azide-resistant variant of SecA (SecAT128A, *azi-1*). Scale bars are 5 μm.

Both *sec341* and *secA12* mutations are located in the intramolecular regulator of ATPase 2 (IRA2) domain of SecA. Since mutations in this region have been shown to severely reduce the ATPase activity of SecA (Sianidis et al., 2001), it is likely that this activity is essential for DivIVA targeting. To test this, cells were treated with sodium azide that specifically inhibits the ATPase activity of SecA (Nakane et al., 1995). Strain 4067 (Δ*divIVA, Pxyl-divIVA-gfp*) was grown until mid-log phase (OD_600_ ~ 0.4) and cells were exposed to increasing concentrations of sodium azide (0 mM, 0.1 mM, 0.5 mM, and 1.0 mM). After 30 min, DivIVA-GFP expression was induced with 0.5% xylose. Two hours later, a typical DivIVA-GFP fluorescence pattern was observed in the absence of azide, however, increasing azide levels led to an increasingly disturbed localization pattern (**Figure 6A**). The Western blot in **Figure 6B** shows that this is not a consequence of proteolytic degradation, although the induction at higher azide concentrations is clearly less efficient. To rule out that the observed effects were the result of an unspecific azide effect, the experiment was repeated with a strain containing the *azi-1* mutation that renders SecA azide resistant (Nakane et al., 1995) (SecAT128A, strain BSN228). As expected, this strain shows normal localization of DivIVA-GFP even in the presence of 0.5 mM sodium azide (**Figures 6C,D**), supporting the conclusion that the ATPase activity of SecA is required for localization of DivIVA.

### EFFECT OF SecY DEPLETION ON DivIVA LOCALIZATION

**FIGURE 7:**
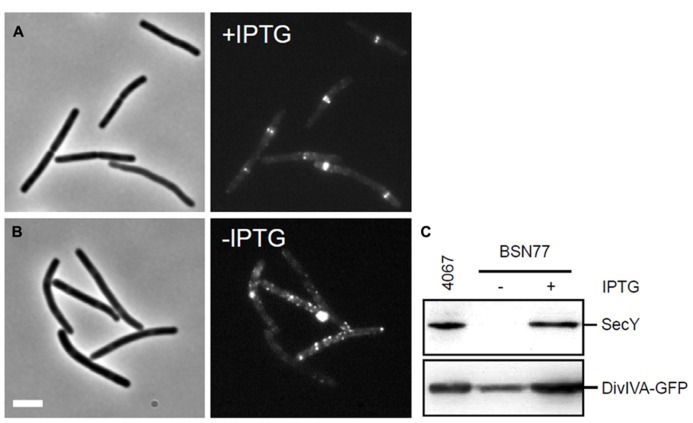
** Effect of SecY depletion on the localization of DivIVA-GFP**. **(A,B)** Localization of DivIVA-GFP in strain BSN77 containing the inducible *Pspac-secY* allele. Strain BSN77 was grown in the presence **(A)** or absence **(B)** of 1 mM IPTG, and expression of *divIVA-gfp* was induced with xylose. The corresponding phase contrast images are presented on the left. Scale bar is 5 μm. **(C)** Western blot analysis to confirm SecY depletion (upper panel), and to monitor the stability of the DivIVA-GFP fusion (lower panel).

SecA delivers proteins to the secretion pore of which the transmembrane protein SecY is a key component (Tjalsma et al., 2000). To examine whether inactivation of the secretion machinery influences DivIVA localization, strain BSN77 was constructed, containing an IPTG-inducible copy of *secY*. BSN77 was grown in the presence of IPTG, and subsequently diluted into fresh medium without IPTG. When the growth rate decreased, indicating depletion of SecY, the expression of DivIVA-GFP was induced with 0.5% xylose. As shown in **Figures 7A,B**, depletion of SecY resulted in spotty and irregular fluorescence pattern. Western blot analyses confirmed the SecY depletion and indicated that this effect was not a result of proteolytic degradation of DivIVA-GFP (**Figure 7C**). These data suggests that localization of DivIVA requires a functional secretion machinery, although this might still be related to effects on SecA activity, as discussed below.

## DISCUSSION

Here we show that the secretion motor protein SecA is important for the localization of DivIVA. Since DivIVA is essential for the subcellular positioning of MinD and RacA, two proteins that are required for efficient sporulation, our finding might partially explain why the *secA12* and *secA341* mutants show an asporogenic phenotype. Both mutations are known to reduce sporulation efficiency 10^2^- to 10^5^-fold (Sadaie and Kada, 1983; Kobayashi et al., 2000), depending on the tested conditions, while loss of *divIVA* results in a 20-fold reduction of spore formation (Thomaides et al., 2001). Thus, besides affecting membrane targeting of DivIVA, SecA has a more pleiotropic effect on endospore formation.

### DIRECT OR INDIRECT ROLE FOR SecA

SecA is the ATPase of the Sec translocon that mediates protein transport across the cell membrane, and membrane integration of transmembrane proteins. The protein recognizes and binds to preproteins via a short N-terminal signal sequence that is later cleaved off by a specific signal sequence peptidase (Tjalsma et al., 2000). SecA unfolds its substrate to thread it through the protein conducting SecYEG channel. This process is driven by repeated cycles of ATP hydrolysis and mechanistically coupled to large conformational changes in SecA and SecYEG (Tsukazaki et al., 2008; Robson et al., 2009). In contrast to a previous report (Campo et al., 2004), SecA is homogenously distributed over the cell membrane (Carballido-Lopez et al., 2006), suggesting that its activity is not restricted to a particular membrane region. Since DivIVA does not have a signal sequence, it seems reasonable to assume that the effects we have observed are indirect, and a consequence of, e.g., changes in cell membrane composition due to perturbed membrane protein insertion. This would explain why SecY is also required for DivIVA localization. However, SecY depletion prevents the transfer of protein substrates from SecA to the secretion pore, as a consequence of which SecA remains occupied and cannot be recycled. SecA also needs to be in contact with SecYEG for full stimulation of its ATPase activity (Lill et al., 1989). Thus, depletion of SecY results in a reduction of SecA activity. The sporulation defect of the *secA12* mutation can be suppressed by compensatory mutations in *secY *(Kobayashi et al., 2000) which also suggests participation of SecY in DivIVA targeting. However, this effect does not reveal whether the role of SecA in DivIVA localization is direct or indirect. A possible change in lipid composition due to SecA inactivation is also unlikely to affect DivIVA binding, since biochemical experiments have shown that purified DivIVA binds directly to lipid bilayers. Moreover, the DivIVA-membrane interaction does not depend on specific lipid species (Lenarcic et al., 2009). A key argument to propose a more direct involvement of SecA in DivIVA localization is the *in vivo* cross-linking data.

### BINDING OF SecA TO DivIVA

The N-terminal domain of DivIVA forms a dimeric coiled–coil structure whereby the amphipathic helices cross each other and expose two phenylalanines that insert into the membrane (Oliva et al., 2010). Mutations that interrupt the hydrophobic interface of the coiled–coil give rise to mislocalized DivIVA (Lenarcic et al., 2009; Oliva et al., 2010). The importance of the N-terminus for the functioning of DivIVA is also illustrated by the fact that this region is phylogenetically most conserved. We have looked for any sequence that shows some resemblance to a signal sequence in the N-terminus of DivIVA. Sec-type signal sequences have an average length of 28 amino acids and start with several positively charged amino acids (N-domain), followed by a hydrophobic core sequence (H-domain), and the signal peptide cleavage site (C-domain; Tjalsma et al., 2000). They adopt an α-helical conformation and interact with SecA via hydrophobic interactions (Gelis et al., 2007). Although the DivIVA N-terminus contains several positively charged amino acids (K11, K15, R18) and forms a helical coil, no distinct H- and C-domain could be predicted using simple sequence comparisons, hydropathy plots or the SignalP3.0 algorithm (Bendtsen et al., 2004). Possibly, the hydrophobic face of the coiled–coil helix in the DivIVA N-terminus is in transient contact with the hydrophobic surface of the SecA signal peptide binding cleft, and therefore substitutes for a signal sequence (Gelis et al., 2007). Interestingly, a recent study showed that DivIVA from *L. monocytogenes* is required for protein translocation via the accessory SecA2-dependent secretion pathway that is present in this organism and involved in virulence (Halbedel et al., 2012). Presently it is unclear how *L. monocytogenes* DivIVA excerts its effect on the SecA2 ATPase, however, based on the cross-linking results it is tempting to speculate that DivIVA homologs have a more general ability to interact with SecA proteins, even though the functional implications of these interactions can be different.

### POSSIBLE FUNCTION OF SecA

The possible function of SecA in DivIVA localization remains speculation. We have tested the addition of purified SecA on the binding efficiency of DivIVA to artificial lipid membranes (Oliva et al., 2010), but were unable to detect any effect. In a previous study it was shown that lipid binding of DivIVA dimers is very weak, and only large oligomeric DivIVA structures give rise to strong membrane binding (Oliva et al., 2010). It is therefore possible that a stimulatory effect of SecA can only be observed *in vivo*, since in cells the cytoplasm and membrane is crowded with proteins and DivIVA might require SecA to be targeted efficiently to the cell membrane. Another possible explanation is that SecA is required for the proper folding of DivIVA. DivIVA is primarily alpha-helical and contains several coiled–coil helices that contribute to the oligomerization of the protein (Rigden et al., 2008; Oliva et al., 2010; van Baarle et al., 2013). Proper folding of DivIVA might require the assistance of chaperones. It has been shown that SecA can act as a chaperone in the refolding of proteins that lack a signal sequence, although this activity did not require ATP (Eser and Ehrmann, 2003). We have tried to restore the lipid binding activity of heat denatured, or urea denatured, DivIVA by incubation with SecA (and ATP) but without success. In addition, using native gel electrophoresis, we also could not detect any effect on DivIVA oligomerization in the *secA341* mutant background.**Finally, it should be added that depletion of SecA might affect the membrane potential, and this can disturb the localization of peripheral membrane proteins (Strahl and Hamoen, 2010). However, it has been shown that the localization of DivIVA does not rely on the presence of the proton motive force (Strahl and Hamoen, 2010).

In conclusion, it remains to be determined why SecA is required for the localization of DivIVA. We cannot rule out the possibility that there is a SecA-dependent membrane protein with a direct function in DivIVA membrane binding. However, our data raises the possibility that SecA might be directly involved in membrane targeting of a peripheral membrane protein that does not contain a clear signal sequence.

## Conflict of Interest Statement

The authors declare that the research was conducted in the absence of any commercial or financial relationships that could be construed as a potential conflict of interest.
